# Supplementary material for: Possible Reduction of Cardiac Risk after Supplementation with Epigallocatechin Gallate and Increase of Ketone Bodies in the Blood in Patients with Multiple Sclerosis. A Pilot Study
Source: Nutrients. 2020 Dec 10;12(12):3792. doi: 10.3390/nu12123792 (PMC7763038; doi:10.3390/nu12123792)
Supplement: Supplementary file 1 [file nutrients-12-03792-s001.pdf]

Supplementary material

Table S1. Intra-group comparison before treatment (pre) and after treatment (post) of cardiovascular risk.

**Control group N=17**		**Pre**		**Post**			
		**Freq**	**%**	**Freq**	**%**	**Chi2**	***p***
**Cardiac risk**	Low	5	29.4%	8	47.1%	-1.342	.180
	Moderate	4	23.5%	1	5.9%		
	High	8	47.1%	8	47.1%		
**Intervention group N=16**		**Pre**		**Post**			
		**Freq**	**%**	**Freq**	**%**	**Chi2**	***p***
Cardiac risk	Low	5	31.3%	6	37.5%	-2.000	.046
	Moderate	2	12.5%	4	25.0%		
	High	9	56.3%	6	37.5%		

Chi2: Chi square test.
